# KAT6B May Be Applied as a Potential Therapeutic Target for Glioma

**DOI:** 10.1155/2022/2500092

**Published:** 2022-04-06

**Authors:** Yingzi Liu, Xiaoyang Duan, Chunyan Zhang, Jiangwei Yuan, Junpeng Wen, Cuihong Zheng, Jian Shi, Meng Yuan

**Affiliations:** ^1^Department of Neurosurgery, The Fourth Hospital of Hebei Medical University, Shijiazhuang, Hebei Province, China; ^2^Department of Medical Oncology, The Fourth Hospital of Hebei Medical University, Shijiazhuang, Hebei Province, China; ^3^Faculty of Medicine, School of Clinical Medicine, Hebei University, Shijiazhuang, Hebei Province, China; ^4^Department of Internal Medicine, University of Occupational and Environmental Health, Fukuoka 807-8555, Japan

## Abstract

Glioma is a prevalent malignancy among brain tumors with high modality and low prognosis. Ferroptosis has been identified to play a crucial role in the progression and treatment of cancers. KAT6B, as a histone acetyltransferase, is involved in multiple cancer development. However, the function of KAT6B in glioma is still elusive. Here, we aimed to evaluate the effect of KAT6B on ferroptosis in glioma cells and explored the potential mechanisms. We observed that the expression of KAT6B was enhanced in clinical glioma samples. The viability of glioma cells was repressed by erastin and the overexpression of KAT6B rescued the phenotype in the cells. Meanwhile, the apoptosis of glioma cells was induced by the treatment of erastin, while the overexpression of KAT6B blocked the effect in the cells. The levels of lipid ROS and iron were promoted by the treatment of erastin and the overexpression of KAT6B could reverse the effect in the cells. Mechanically, we identified that the expression of STAT3 was repressed by the KAT6B knockdown in glioma cells. The KAT6B was able to enrich on the promoter of STAT3 in glioma cells. Meanwhile, ChIP assay showed that the knockdown of KAT6B inhibited the enrichment of histone H3 lysine 23 acetylation (H3K23ac) and RNA polymerase II (RNA pol II) on STAT3 promoter in the cells. Depletion of STAT3 reversed KAT6B-regulated viability, apoptosis, and ferroptosis of glioma cells. Thus, we concluded that KAT6B contributes to glioma progression by repressing ferroptosis *via* epigenetically inducing STAT3.

## 1. Introduction

Glioma serves as a prevalent malignancy among brain tumors in adults, which is also highly aggressive and with grave prognosis [1]. Cancer development is a complicated process resulting from various aspects regarding proliferation, apoptosis, angiogenesis, and especially metabolism [2]. Over the past decade, as a distinctive kind of RCD, ferroptosis is initially recognized while a small molecule termed erastin can constrain the potential of triggering an RCD process, particularly in RAS-mutated cancer cells [3]. The introduction of ferroptosis causes the interference of cancer progression, and the ferroptosis inducers in repressing tumor cells and decreasing tumor growth can be served as potential antitumor treatments [4].

KAT6B is a major histone acetyltransferase, widely reported during the progression of various diseases, including cancer, cardiovascular diseases, and neurodegenerative diseases [5]. KAT6B exhibits diverse functions in eukaryotes, especially the participation in regulation of RNA transcription, autophagy, DNA repair, proteasome-dependent protein turnover, circadian rhythms, learning and memory, and neurological functions associated with aging and neurodegeneration [6, 7]. Previous studies have presented the roles of KAT6B in cancer development. It has been reported that microRNA-4513 enhances epithelial-mesenchymal transition and cell proliferation of gastric cancer by modulating KAT6B [8]. However, the effect of KAT6B on glioma is still unreported. Meanwhile, STAT3, as a crucial transcription factor, plays crucial oncogene roles in glioma. For example, it has been reported that STAT3 contributes to tumor progression by promoting FOXP1 transcription in glioma [9]. miR-519a improves chemosensitivity and enhances autophagy in glioma by targeting STAT3/Bcl2 signaling [10]. Hypoxic glioma-derived exosomes deliver miRNA-1246 to promote M2 macrophage polarization by regulating TERF2IP through STAT3 signaling [11]. KAT6B is able to regulate histone H3 lysing 23 acetylation (H3K23ac), which is associated with the regulation of STAT3 [12]. However, the effect of KAT6B on STAT3 remains elusive.

In this work, we studied the function of KAT6B in glioma cells. We aimed to evaluate the effect of KAT6B on ferroptosis in glioma cells and explored the potential mechanisms.

## 2. Materials and Methods

### 2.1. Clinical Samples

The glioma samples (*n* = 35) were collected from glioma patients hospitalized, who received no chemotherapy and radiotherapy before surgery. All tumor samples were clinicopathologically confirmed as glioma (6 grade I, 7 grade II, 10 grade III, and 12 grade IV). All of glioma tissues were astrocytoma and IDH wildtype. Normal brain tissues were collected from patients undergoing brain tissue resection due to craniocerebral injury. All samples were immediately stored in liquid nitrogen with RNAhold (TransGen). All patients have signed the informed consent forms. All experiments were approved by the Clinical Ethics Committee of our hospital.

### 2.2. Cell Culture

Human glioma cell lines U251 and LN229 were purchased from the American Type Culture Collection (ATCC, USA), maintained in DMEM medium (Hyclone, USA) added with 10% FBS (Gibco, USA) and 1% penicillin/streptomycin (Sigma, USA), and were incubated in a 37°C atmosphere with 5% CO_2_.

### 2.3. Cell Transfection

The small interference RNA targeting KAT6B and STAT3 was designed and synthesized by Ribobio (China). Recombinant plasmid pcDNA-KAT6B was constructed by cloning KAT6B cDNA into pcDNA3.1 vectors (Ribobio, China). The U251 and LN229 cells were seeded in a 6-well plate with 4 × 10^5^ cells per well and transfected with 50 nmol oligonucleotides or their corresponding negative controls [7] by using transfection reagent Lipofectamine 2000 (Thermo, USA) according to manufacturer's instruction.

### 2.4. Cell Viability and Apoptosis

Cell viability was evaluated by the cell counting kit-8 (CCK-8, Thermo, USA) in accordance with the manufacturer's description. The cells were planted in a 96-well plate, incubated overnight, and treated with corresponding oligonucleotides. At indicated time point, the CCK-8 solution (100 *μ*l/well) was added to each well and incubated for another 2 h at 37°C. The absorbance values were measured at 450 nm using a microplate detector (BioRad, USA).

### 2.5. Colony Formation Assays

About 1 × 10^3^ cells were plated in 6-well dishes and cultured in DMEM at the condition of 5% CO_2_ and 37°C. After 2 weeks, cells were cleaned with PBS Buffer, made in methanol about thirty minutes, and dyed with crystal violet dye at the dose of 1%, after which the number of colonies was calculated.

### 2.6. Ferroptosis Analysis

The lipid ROS was measured by flow cytometry analysis in the cells. For Lipid ROS, cells were treated as indicated, and then typsinezed and resuspended in medium plus 10% FBS. Then, 10 *μ*M of C11-BODIPY (Thermo Fisher, Cat# D3861) was added, and samples were incubated for 30 min at 37°C, 5% CO2 and protected from light. Excess C11-BODIPY was removed by washing the cells twice with PBS. Oxidation of the polyunsaturated butadienyl portion of the dye results in a shift of the fluorescence emission peak from 590 nm to 510 nm in a manner proportional to lipid ROS generation. This shift was analyzed using a flow cytometer [13]. The levels of iron were analyzed by Iron Assay Kit in the cells. For the iron assay, we used the Iron Assay Kit (Sigma Aldrich) to measure the Fe^2+^ or total iron in each cell line. 2 × 10^6^ of cells were rapidly homogenized in 4-10 volumes of Iron Assay buffer. Samples were centrifuged at 13,000 g for 10 minutes at 4°C to remove insoluble material. To measure ferrous iron, 5 *μ*L of iron assay buffer was added to each well. To measure ferric iron, two sets of wells were set up. Then, 5 *μ*L of assay buffer was added to the samples in one set of wells and 5 *μ*L of iron reducer was added to the other set of wells. To measure total iron, add 5 *μ*L of Iron Reducer to each of the sample wells to reduce Fe3+ to Fe2+. Samples were mixed well using a horizontal shaker or by pipetting, and the reactions were incubated for 30 minutes at room temperature, protected from light. Then, 100 *μ*L of Iron Probe was added to each well-containing standard or test samples. Samples were mixed well using a horizontal shaker or by pipetting, and the reactions were incubated for 60 minutes at room temperature, protected from light. Finally, the absorbance was measured at 593 nm (A593) [13].

### 2.7. Quantitative Real-Time PCR (qRT-PCR)

Total RNAs were isolated from tissues or cells using Trizol reagent (Thermo, USA), reversely transcribed into cDNA by using the High Capacity cDNA Reverse Transcription Kit (Thermo, USA). The relative RNA levels were quantified by quantitative real-time PCR using the SuperScript III Platinum SYBR Green One-Step qRT-PCR Kit (Thermo, USA). GAPDH was adopted as internal control for normalization following the 2^-*ΔΔ*Ct^ method.

The primer sequences are as follows: KAT6B forward: 5′- ATACGAGCGAATGGGTCAGAGTGATTTTGG-3′, reverse: 5′- GTTCACAGAGTTGACATTGTAGGCTGGCG-3′; STAT3 forward: 5′- AACTCTCACGGACGAGGAGCT-3′, reverse: 5′-AGTAGTGAACTGGACGCCGG-3′; GAPDH forward: 5′-AACGGATTTGGTCGTATTGGG-3′, reverse: 5′-CCTGGAAGATGGTGATGGGAT-3′.

### 2.8. Chromatin Immunoprecipitation (ChIP) Assay

ChIP assay was performed by using Pierce Magnetic ChIP Kit (Thermo, USA) in accordance with the manufacturer's protocols. Antibodies against KAT6B, H3K23ac, and RNA polymerase II were used to precipitate the DNA-protein complex. The level of immunoprecipitated DNA was evaluated by qRT-PCR assay. Briefly, ChIP assays were essentially performed as previously described [13] with slight modifications: 5 × 10^6^ cells were fixed with formaldehyde (1% final volume concentration, Sigma) for 10 min at room temperature. Fixation was stopped with the addition of 1/10 volume 1.25 M glycine, and the samples were incubated for 5 min at room temperature. The sonication step was performed in a Qsonica sonicator (5 min, 20 s on, 20 s off), and 200 *μ*g of the protein-chromatin complex was used in each immunoprecipitation. Antibody-protein complexes were captured with preblocked dynabeads protein G (Invitrogen, USA). ChIP DNA was analyzed by qPCR with SYBR Green (Biorad) on an ABI-7500 (Applied Biosystems) using the primers specified. The antibodies used are as follows: KAT6B (Abcam, USA), H3K23ac (Active motif), RNA pol II (Abcam, USA), and normal mouse IgG (12-371, Millipore). The promoter sequence (Genome = hg38; chr17-: 4238994242388255; TSS = 42388495; Upstream = 1447, Downstream = 240; Length = 1688) was shown as follows:

TGCCCTGTAGATGCCTCTGTCCCTCATTGGATATGAGGTGGTACGAGCGGTCTGAATCTGTAGACTTAGACAGGCTTCAGGTATCTGGGGAACCATGTGAGTAAAACCTTGTATTGTTTCTGGGGCTAGAATCTAGTCTCCTTCAAACACTCAGAAGGATCTGTGACTGCCTTCAAAGGTTAAGAGTCATTGATTTTTCTCACTAGAAATTAATAAGAACAGCTATCCTTGGGGAGAAGGAATGATGGGGGTAGGGAAAGATTTTCATTTAATATTTTTGACCTTTCTGAACGGTCTGCATTTTCTAAACAGGAACATGTATTAGGTTCATTGAAAAAAAATATGTCAGGGGTTAGCTGAGCAGTGAGATAATGGGTCCTTTTTAGATTTTTGTCTTTATACCTGTTCGTATTAAACAAACACAAATATTAATAACATATTTTTAAAGACCCAAAAAAATAAAAATTAAAAACCCTGATAGTATCAGCACATACACAGAAATCACTCCATTATGCAAAGTTCATCCTCTATTATGAAAGGCAAAATGTCTACATTTCCTATCAACCACTGGCTTCAATTCAGTAAAACTTGCATACCAAGTAGGCAAGGTGGAAAAGAAAAAGGCAGAACATTTCATGTATTTCAATTCAGACGCATAAAAATGTCAAGCCCTACACGTTATCAGCTTTCGTATACACCGTCTTCTGCATTCGCCTGTACGGGCCAATGGGCTAGCTGGTCGGCGTTTGATGCTTGAAGTGATGGAACGGAGTACGGGGTTAAATCCACTACCCTCTCCCCACGCACTCTAGTAATTACTCTATTTCCACGTCATGTTTCCGGGTGTGTGTGTCCCTGCTCACGCAGAAACTGAAGTTCAAAGCAGGCGGAGTCACCCATGTTCTTTTTGTTGTCCCCAGAACCCAATTCAGGAGTTGGGTCCCCAGAGGATCTGGAGATACCTGGGGACTATCTAACTAGCTGATTCCCGCGTGGTAAGAGGCTCTCAACCTCGCCACCACGTGGTGCCAAGGGCCGGGAAAAGGGAGAGCGGGCAGGAGGGAGCTGTATCAGGGGCATTTAAAGTGCCTTGACGTCACGCACTGCCAGGAACTCAGCTGAGTTTTCAGCAGGACATTCCGGTCATCTTCCCTCCCTCCCCCCGGGCTTCTGTGCCCAAGTCCTCGGCTCTTCCCTCGCTGTGGCGGAGGGAGGAGCACCGAACTGTCGGAACAGCCAGCACAGGGGCGTATCAGTCTCCTCTTGGCTCCGCCCTTTCTCCTAGCTGCTCTCCTCATTGGTCAGTGGGCGGGGCTTCGGCTGTACCGCACACGCACTGGGACCTCTGGGTGGCCGAACGAGCTGGCCTTTCATGAATTATGCATGACGGCGTGCCTCGGCCAGGCTGGGGCTGGGCGAGGATTGGCTGAAGGGGCTGTAATTCAGCGGTTTCCGGAGCTGCGGCGGCGCAGACTGGGAGGGGGAGCCGGGGGTTCCGACGTCGCAGCCGAGGGAACAAGCCCCAACCGGATCCTGGACAGGCACCCCGGCTTGGCGCTGTCTCTCCCCCTCGGCTCGGAGAGGCCCTTCGGCCTGAGGGAGCCTCGCCGCCCGTCCCCGGCACACGCGCAGCCCCGGCCTCTCGGCCTCTGCCGGAGAAACAGGTGAAGGGGGTGCAGGGTGGGGCC.

### 2.9. Data Analysis

Data in this work are presented as mean ± SD. Statistical analysis was performed by using SPSS software (version 17.0). Statistical comparison between two or more groups was determined by student's *t* test or one-way ANOVA test. A *p* value less than 0.05 was considered to be statistically significant.

## 3. Results

### 3.1. KAT2B Is Elevated in the Clinical Glioma Samples

**Figure 1:**
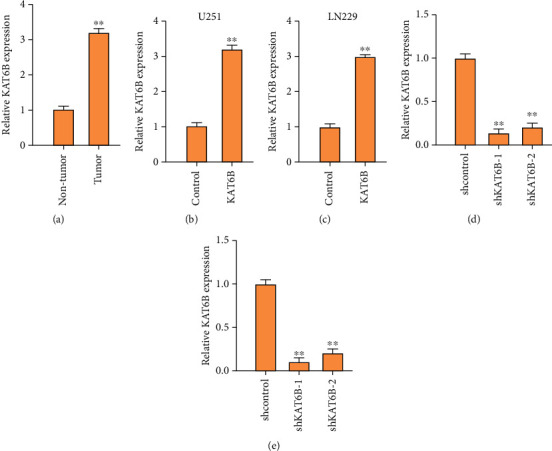
KAT2B is elevated in the clinical glioma samples. (a) The expression of KAT2B was measured by qPCR in clinical glioma tissues (*n* = 35). (b, c) The U251 and LN229 cells were treated with KAT2B overexpressing plasmids, and the expression of KAT2B was detected by qPCR in the cells. Empty plasmid was served as the control. (d, e) The U251 and LN229 cells were treated with shRNAs, and the expression of KAT2B was analyzed by qPCR in the cells. Lentivirus vector containing negative control RNAi sequence was served as shcontrol. ∗∗*p* < 0.01.

To analyze the correlation of KAT2B with glioma, the expression of KAT2B was analyzed in the clinical glioma samples. We observed that the expression of KAT2B was enhanced in clinical glioma samples (Figure 1(a)). To evaluate the effect of KAT2B on glioma, the glioma cell lines, including U251 and LN229 cells, were treated with KAT2B overexpressing plasmids or KAT2B shRNAs, and the effectiveness of KAT2B overexpression and depletion was validated in the cells (Figures 1(b)–1(e)).

### 3.2. The Overexpression of KAT6B Reverses Ferroptosis Activator Erastin-Regulated Viability and Apoptosis of Glioma Cells

**Figure 2:**
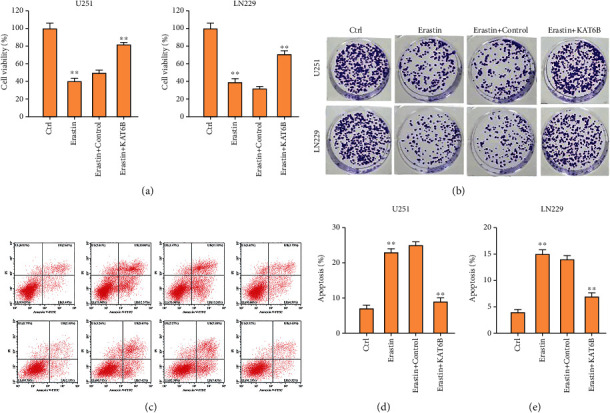
The overexpression of KAT6B reverses ferroptosis activator erastin-regulated viability and apoptosis of glioma cells. (a)–(e) The U251 and LN229 cells were treated with ferroptosis activator erastin (5 *μ*M) for 24 hours or treated with erastin (5 *μ*M) for 24 hours followed by the treatment of KAT6B overexpressing plasmid. Cell viability (a) and colony formation number (b) and apoptosis (c)–(e) were analyzed by CCK-8, colony formation assay, and flow cytometry. ∗∗*p* < 0.01.

Next, we were interested in the correlation of KAT2B with ferroptosis in glioma cells. To this end, the U251 and LN229 cells were treated with ferroptosis activator erastin or cotreated with erastin and KAT6B overexpressing plasmid. We found that the viability and colony formation numbers of U251 and LN229 cells were repressed by erastin and the overexpression of KAT6B rescued the phenotype in the cells (Figures 2(a) and 2(b)). Meanwhile, the apoptosis of U251 and LN229 cells was induced by the treatment of erastin, while the overexpression of KAT6B blocked the effect in the cells (Figures 2(c)–2(e)).

### 3.3. The Overexpression of KAT6B Represses Ferroptosis of Glioma Cells

**Figure 3:**
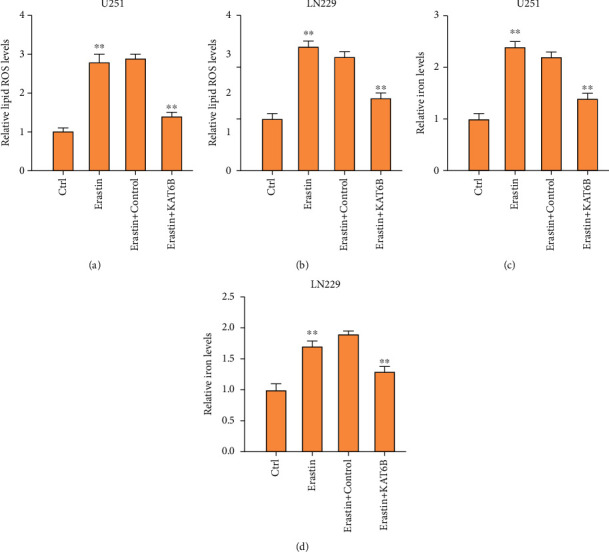
The overexpression of KAT6B represses ferroptosis of glioma cells. (a)–(d) The U251 and LN229 cells were treated with ferroptosis activator erastin (5 *μ*M) for 24 hours or treated with erastin (5 *μ*M) for 24 hours followed by the treatment of KAT6B overexpressing plasmid. The levels of lipid ROS (a, b) and iron (c, d) were analyzed in the cells. ∗∗*p* < 0.01.

Then, we evaluated the effect of KAT6B on the levels of ferroptosis markers, including lipid ROS and iron, in the glioma cells. We observed that the levels of lipid ROS and iron were promoted by the treatment of erastin and the overexpression of KAT6B could reverse the effect in the cells (Figures 3(a)–3(d)).

### 3.4. KAT6B Epigenetically Promotes STAT3 Expression in Glioma Cells

**Figure 4:**
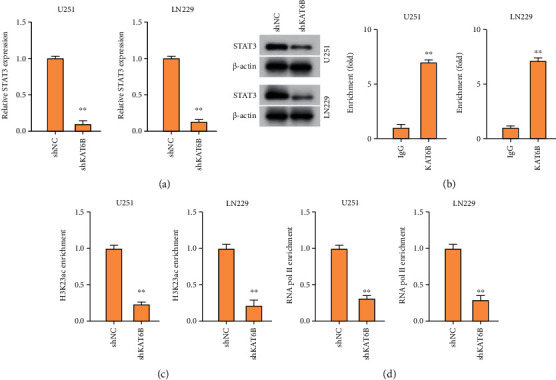
KAT6B epigenetically promotes STAT3 expression in glioma cells. (a) The expression of STAT3 was measured by RT-PCR assay and Western blot analysis in U251 and LN229 cells treated with shKAT6B. (b) The enrichment of KAT6B on STAT3 promoter was analyzed by ChIP assay. (c, d) The U251 and LN229 cells were treated with shKAT6B. The enrichment of H3K23ac (c) and RNA pol II (d) on STAT3 promoter was analyzed by ChIP assay. ∗∗*p* < 0.01.

We next tried to explore the underlying mechanism by which KAT6B regulated glioma. We observed that the expression of STAT3 was repressed by the KAT6B knockdown in U251 and LN229 cells (Figure 4(a)). We identified that the KAT6B was able to enrich on the promoter of STAT3 in U251 and LN229 cells (Figure 4(b)). Meanwhile, ChIP assay showed that the knockdown of KAT6B inhibited the enrichment of histone H3 lysine 23 acetylation (H3K23ac) and RNA polymerase II (RNA pol II) on STAT3 promoter in U251 and LN229 cells (Figures 4(c) and 4(d)).

### 3.5. Depletion of STAT3 Reverses KAT6B-Regulated Viability, Apoptosis, and Ferroptosis of Glioma Cells

**Figure 5:**
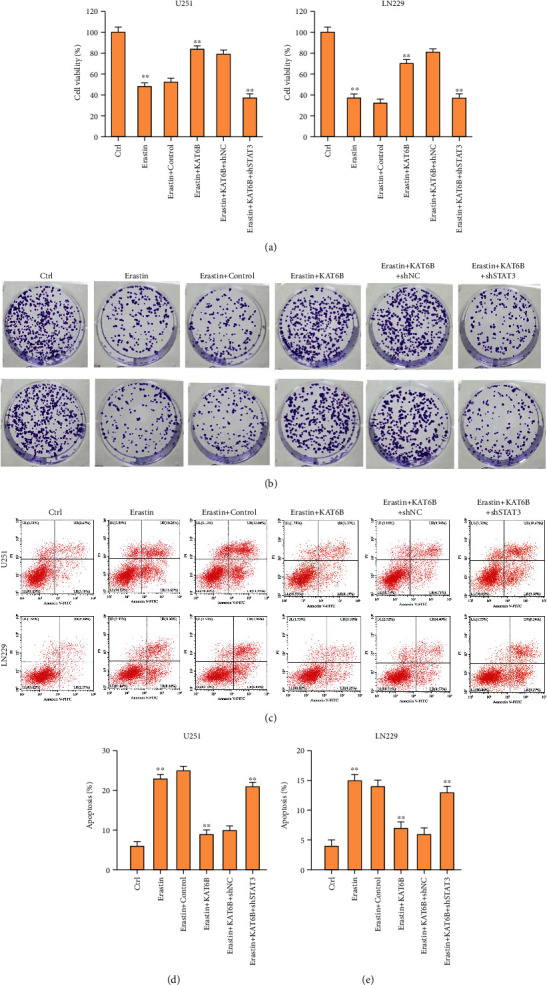
Depletion of STAT3 reverses KAT6B-regulated viability and apoptosis of glioma cells. (a)–(e) The U251 and LN229 cells were treated with ferroptosis activator erastin (5 *μ*M) for 24 hours or treated with erastin (5 *μ*M) for 24 hours followed by the treatment of KAT6B overexpressing plasmid and STAT3 shRNA. Cell viability (a) and colony formation number (b) and apoptosis (c)–(e) were analyzed by CCK-8, colony formation assay, and flow cytometry. ∗∗*p* < 0.01.

**Figure 6:**
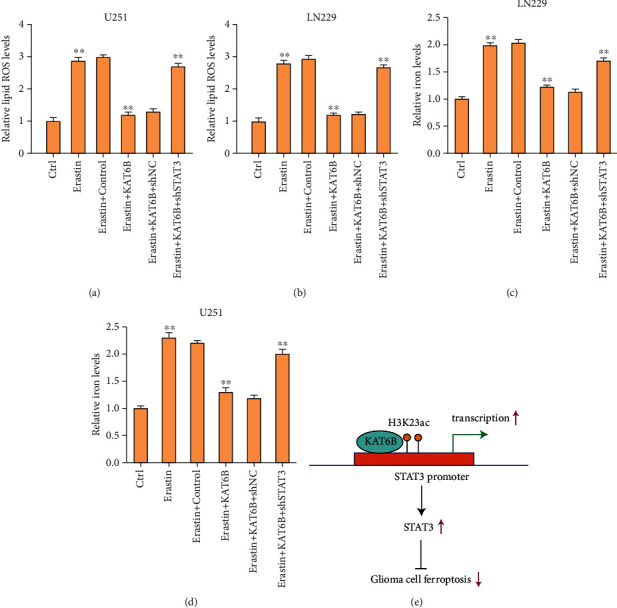
Depletion of STAT3 reverses KAT6B-regulated ferroptosis of glioma cells. (a)–(d) The U251 and LN229 cells were treated with ferroptosis activator erastin (5 *μ*M) for 24 hours or treated with erastin (5 *μ*M) for 24 hours followed by the treatment of KAT6B overexpressing plasmid and STAT3 shRNA. The levels of lipid ROS (a, b) and iron (c, d) were analyzed in the cells. (e) A schematic diagram was shown. ∗∗*p* < 0.01.

Next, we validated the correlation of KAT6B and STAT3 in the regulation of glioma cells. The effectiveness of STAT3 depletion by shRNA was validated in U251 and LN229 cells (Figure S1). We observed that the viability and colony formation numbers of U251 and LN229 cells were repressed by erastin, and the overexpression of KAT6B rescued the phenotype in the cells, in which the depletion of STAT3 could referee the effect of KAT6B (Figures 5(a) and 5(b)). Meanwhile, the apoptosis of U251 and LN229 cells was enhanced by the treatment of erastin, and the overexpression of KAT6B blocked the effect in the cells, while the depletion of STAT3 could reverse the effect (Figures 5(c)–5(e)). The levels of lipid ROS and iron were induced by the treatment of erastin, and the overexpression of KAT6B repressed the levels in the cells, in which the depletion of STAT3 could reverse the effect of lipid (Figures 6(a)–6(d)).

## 4. Discussion

Glioma is a prevalent malignancy among brain tumors, and ferroptosis plays crucial roles in the progression and treatment of cancers. Histone acetyltransferase KAT6B is involved in multiple cancer development but the function of KAT6B in glioma remains unclear. In this study, we uncovered the effect and the potential mechanism of KAT6B on ferroptosis in glioma cells.

Previous studies have identified the function of KAT6B in cancer development. The inhibition of histone acetyltransferases KAT6A/B contributes to senescence and regulates tumor growth [14]. KAT6B functions as a tumor suppressor to modulate histone H3 lysine 23 acetylation in genomic loss in small cell lung cancer [15]. miR-22/KAT6B axis serves as a chemotherapeutic regulator by the modulation of PI3k/Akt/NF-*κ*B signaling in tongue squamous cell carcinoma [16]. These reports indicate that KAT6B may play oncogenic or tumor inhibitory function in different cancers. In the current work, we found that the expression of KAT2B was enhanced in clinical glioma samples. The viability of glioma cells was repressed by erastin, and the overexpression of KAT6B rescued the phenotype in the cells. Meanwhile, the apoptosis of glioma cells was induced by the treatment of erastin, while the overexpression of KAT6B blocked the effect in the cells. The levels of lipid ROS and iron were promoted by the treatment of erastin, and the overexpression of KAT6B could reverse the effect in the cells. These data suggest that KAT6B represses ferroptosis of glioma cells. The clinical significance of KAT6B should be explored in future investigations. In this study, overexpression of KAT6B could inhibit both apoptosis and ferroptosis of glioma cells. The relationship of KAT6B-mediated apoptosis and ferroptosis and what is the main mechanism of KAT6B promoting glioma malignancy should be explored in future investigations.

Moreover, it has been reported that STAT3 inhibits ferroptosis-mediated IIR-ALI by targeting SLC7A11 [17]. miR-519a promotes chemosensitivity and enhances autophagy of glioma cells by regulating STAT3/Bcl2 signaling [10]. STAT3 contributes to glioma progression by promoting FOXP1 transcription [9]. Here, we identified that the expression of STAT3 was repressed by the KAT6B knockdown in glioma cells. The KAT6B was able to enrich on the promoter of STAT3 in glioma cells. Meanwhile, ChIP assay showed that the knockdown of KAT6B inhibited the enrichment of histone H3 lysine 23 acetylation (H3K23ac) and RNA polymerase II (RNA pol II) on STAT3 promoter in the cells. Depletion of STAT3 reversed KAT6B-regulated viability, apoptosis, and ferroptosis of glioma cells. Our finding provides novel insights into the mechanism by which KAT6B regulates glioma progression by STAT3. There were still some deficiencies in this study. For example, the clinical significance and correlation of KAT6B and STAT3 should be explored by more studies. Meanwhile, STAT3 may be just one of the downstream factors of KAT6B-mediated glioma progression, and other mechanisms should be identified in future investigations.

## 5. Conclusion

Therefore, we concluded that KAT6B contributes to glioma progression by repressing ferroptosis *via* epigenetically inducing STAT3 (Figure 6(e)). KAT6B may be applied as a potential therapeutic target for glioma.

## Data Availability

The datasets used and/or analyzed during the current study are available from the corresponding author on reasonable request.

## Supplementary Materials

Supplementary Materials Figure S1: the validation of STAT3 depletion. (a) The U251 and LN229 cells were treated with STAT3 shRNA, and the expression of STAT3 was analyzed by qPCR in the cells. Lentivirus vector containing negative control RNAi sequence was served as shcontrol. ∗∗*p* < 0.01.
