# Supplementary figures and images for: KAT6B May Be Applied as a Potential Therapeutic Target for Glioma
Source: J Oncol. 2022 Apr 6;2022:2500092. doi: 10.1155/2022/2500092 (PMC9007634; doi:10.1155/2022/2500092)

**Figure S1**

**A**

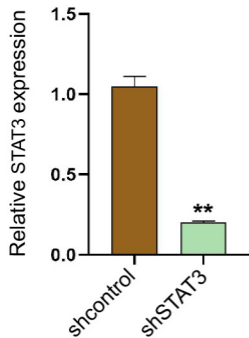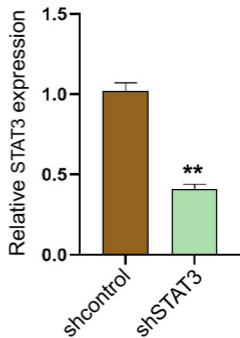

Supplement: Supplementary Materials — Figure S1: the validation of STAT3 depletion. (a) The U251 and LN229 cells were treated with STAT3 shRNA, and the expression of STAT3 was analyzed by qPCR in the cells. Lentivirus vector containing negative control RNAi sequence was served as shcontrol. ∗∗p < 0.01. [file 2500092.f1.pdf]
